# Prophet forecasting model: a machine learning approach to predict the concentration of air pollutants (PM_2.5_, PM_10_, O_3_, NO_2_, SO_2_, CO) in Seoul, South Korea

**DOI:** 10.7717/peerj.9961

**Published:** 2020-09-15

**Authors:** Justin Shen, Davesh Valagolam, Serena McCalla

**Affiliations:** Department of Science Research, Jericho Senior High School, Jericho, NY, United States of America

**Keywords:** Prophet forecasting model, Air pollution, Seoul, South Korea, Particulate matter, Carbon monoxide, Sulfur dioxide, Nitrogen dioxide, Tropospheric ozone, Prediction model

## Abstract

Amidst recent industrialization in South Korea, Seoul has experienced high levels of air pollution, an issue that is magnified due to a lack of effective air pollution prediction techniques. In this study, the Prophet forecasting model (PFM) was used to predict both short-term and long-term air pollution in Seoul. The air pollutants forecasted in this study were PM_2.5_, PM_10_, O_3_, NO_2_, SO_2_, and CO, air pollutants responsible for numerous health conditions upon long-term exposure. Current chemical models to predict air pollution require complex source lists making them difficult to use. Machine learning models have also been implemented however their requirement of meteorological parameters render the models ineffective as additional models and infrastructure need to be in place to model meteorology. To address this, a model needs to be created that can accurately predict pollution based on time. A dataset containing three years worth of hourly air quality measurements in Seoul was sourced from the Seoul Open Data Plaza. To optimize the model, PFM has the following parameters: model type, changepoints, seasonality, holidays, and error. Cross validation was performed on the 2017–18 data; then, the model predicted 2019 values. To compare the predicted and actual values and determine the accuracy of the model, the statistical indicators: mean squared error (MSE), mean absolute error (MAE), root mean squared error (RMSE), and coverage were used. PFM predicted PM_2.5_ and PM_10_ with a MAE value of 12.6 µg/m^3^ and 19.6 µg/m^3^, respectively. PFM also predicted SO_2_ and CO with a MAE value of 0.00124 ppm and 0.207 ppm, respectively. PFM’s prediction of PM_2.5_ and PM_10_ had a MAE approximately 2 times and 4 times less, respectively, than comparable models. PFM’s prediction of SO_2_and CO had a MAE approximately five times and 50 times less, respectively, than comparable models. In most cases, PFM’s ability to accurately forecast the concentration of air pollutants in Seoul up to one year in advance outperformed similar models proposed in literature. This study addresses the limitations of the prior two PFM studies by expanding the modelled air pollutants from three pollutants to six pollutants while increasing the prediction time from 3 days to 1 year. This is also the first research to use PFM in Seoul, Korea. To achieve more accurate results, a larger air pollution dataset needs to be implemented with PFM. In the future, PFM should be used to predict and model air pollution in other regions, especially those without advanced infrastructure to model meteorology alongside air pollution. In Seoul, Seoul’s government can use PFM to accurately predict air pollution concentrations and plan accordingly.

## Introduction

### Air pollution in Seoul, South Korea

Global air pollution is the leading environmental cause of death estimated to contribute to approximately 14 million deaths annually, while leading to over 3.2% of global disease (Babatola, 2018; North et al., 2019). The recent industrial expansion in numerous Asian countries has led to a subsequent spike in air pollution (North et al., 2019). In South Korea, air pollution has already demonstrated significant impacts on human health (AQLI, 2019). On average, South Korean citizens are expected to have a 1.4 year decrease in lifespan due to air pollution levels exceeding World Health Organization (WHO) standards (AQLI, 2019). For those who live in the capital of South Korea, Seoul, life expectancy is predicted to decrease by 1.7 years due to air pollution (AQLI, 2019).

### Air pollution impacts on human and economic health

Key air pollutants in South Korea include particulate matter less than 2.5 µm (PM_2.5_), particulate matter less than 10 µm (PM_10_), ozone (O_3_), nitrogen dioxide (NO_2_), sulfur dioxide (SO_2_), and carbon monoxide (CO). PM_2.5_ and PM_10_ severely degrade human respiratory health leading to increased mortality and morbidity (Xing et al., 2016). PM_2.5_ and PM_10_ are composed of free radical inducing substances, such as transition metals (copper, zinc, manganese), polycyclic aromatic hydrocarbons, and lipopolysaccharide, leading to accelerated respiratory degradation (Xing et al., 2016). In a case study of 5 European countries, the presence of PM_2.5_ was correlated to a 8.6 month decrease in life expectancy (Xing et al., 2016). In another case study investigating 29 European countries, a 10µg/m3 increase in PM_10_ leads to a 0.58% increase in respiratory-induced mortality (Xing et al., 2016).

Although stratospheric O_3_ is a necessity to life on earth, its harmful counterpart tropospheric O_3_ is a reactive respiratory irritant linked with a variety of respiratory issues, such as a loss of lung capacity and permanent lung scarring (Silverman, 1979). Tropospheric O_3_ is also responsible for a decline in global agricultural yields agricultural damage due to its high phytotoxicity (Comis, 2011; Emberson et al., 2018). Tropospheric O_3_ is estimated to induce up to a 16% loss in agricultural staples like wheat, maize, rice, and soybean (Emberson et al., 2018).

NO_2_ and SO_2_ are dangerous air pollutants formed from the excessive combustion of nitrogen and sulfur-containing fossil fuels (Salonen, Salthammer & Morawska, 2019; Al-Naimi, Balakrishnan & Goktepe, 2015; Chen et al., 2007; Liu et al., 2016). NO_2_ and SO_2_ are severe respiratory irritants implicated in the aggravation of asthma and respiratory inflammation (Salonen, Salthammer & Morawska, 2019; Al-Naimi, Balakrishnan & Goktepe, 2015; Chen et al., 2007; Liu et al., 2016). Both NO_2_ and SO_2_ are also highly reactive with other compounds forming additional environmental pollution, such as PM_2.5_, PM_10_, and acid rain (Salonen, Salthammer & Morawska, 2019; Al-Naimi, Balakrishnan & Goktepe, 2015; Chen et al., 2007; Liu et al., 2016). NO_2_ is also a critical reactant in the chemical processes that form O_3_ and photochemical smog (Salonen, Salthammer & Morawska, 2019; Al-Naimi, Balakrishnan & Goktepe, 2015).

CO is a highly lethal air pollutant that is formed via the complete combustion reaction of fossil fuels (Ely, Moorehead & Haponik, 1995). CO is a highly reactive compound that prevents the absorption of oxygen into the blood leading to dizziness, confusion, and, at high concentrations, death (Graber et al., 2007).

### Limitations in current air pollution prediction models

Current non-machine learning air pollution prediction models have major flaws that prevent their widespread implementation to make long-term predictions. Models, such as the Weather Research and Forecasting Model coupled with Chemistry (WRF-Chem) and the Community Multiscale Air Quality Modelling system (CMAQ), are severely disadvantaged due to their complex source list that requires frequent updates and inability to accurately model air pollution across different terrains; this prevents scalability across different regions, especially those that lack the resources to maintain complicated source lists (Xi et al., 2015; Deters et al., 2017). This has led to numerous studies focusing on the premise of machine learning in air pollution prediction models. Furthermore, Deters et al. (2017) and Li et al. (2015) hypothesized the use of models that rely heavily on meteorological parameters. However, these models are unable to make accurate long-term air pollution predictions as a result of the inability to accurately predict meteorological events, something that is growing in difficulty amidst the climate change crisis (Scher & Messori, 2019). Basing the prediction of air pollution off a separate dynamic variable like meteorology drastically reduces the accuracy of air pollution forecasting. Furthermore, it also decreases the versatility as it requires the modelled region to have advanced infrastructure to track and model meteorological factors; this prevents the model’s use in less-developed countries.

Artificial neural networks (ANNs) have also been used for the prediction of short-term air pollution prediction (Maleki et al., 2019; Pasero & Mesin, 2010). Both Maleki et al. (2019) and Pasero & Mesin (2010) utilized an ANN in the prediction of air pollution. However, previous literature has highlighted the deficiencies of ANNs: ANNs rely on meteorological factors and tend to overfit the data making the architecture of ANNs unfit for time series modelling (Lu & Wang, 2005). As a result, ANNs have been equipped with a feed-forward neural network (FFNN), an addition that still fails to address the deficiencies of ANNs effectively preventing the accurate forecasting of air quality (Freeman et al., 2018).

Similar machine learning algorithms have been developed with Random Forest Regression (RFR) (Kamińska, 2018). RFR is a popular approach due to its nonlinear pattern, which requires the tuning of a few parameters (Ivanov et al., 2018). However, RFR cannot interpolate data and its maximum and minimum values are bound to its highest and lowest training set data limiting the accuracy of its predictions.

Additionally, Fuller, Carslaw & Lodge (2002) utilized a model to predict PM_10_ concentrations in London based on PM_10_ - NO_*x*_ relationships. However, this model relied heavily on the dynamic PM_10_ - NO_*x*_ relationship that is predicted to change in the future amidst the development of new regulations and technologies (Fuller, Carslaw & Lodge, 2002). This model is also specific for PM_10_ and does not demonstrate the potential to be applied to other important air pollutants (Fuller, Carslaw & Lodge, 2002).

### Prophet Forecasting Model (PFM) as a versatile and accurate prediction tool

PFM is a forecasting model developed by Facebook, which forecasts a desired variable with respect to time (Taylor & Letham, 2017). PFM’s novel ability to forecast accurately without a plethora of complex parameters (meteorology) drastically increases the versatility and applications of PFM (Taylor & Letham, 2017). Due to the varying availability of meteorological data across regions, modelling air pollution with respect to meteorology is unrealistic and unscalable; this prompts the use of PFM as PFM’s accuracy does not rely on the accuracy of other predictive models, such as those used for meteorology (Taylor & Letham, 2017). PFM is also able to operate effectively even if the dataset has numerous outliers and missing values, a characteristic that is important in air pollution modelling as air quality stations often have technological breakdowns that lead to outliers and missing data (Ye, 2019). Furthermore, PFM has demonstrated success over other widely accepted models, such as the autoregressive integrated moving average (ARIMA) and seasonal autoregressive integrated moving average model (SARIMA) in the prediction of air pollution (Ye, 2019; Samal et al., 2019). In fact, PFM takes approximately 10 times less time to train than the comparable ARIMA model (Ye, 2019). PFM’s unique ability to model air pollution accurately without the use of meteorological parameters makes it a preferred method to predict air pollution over current approaches. Only a few studies have utilized PFM for air pollution modelling (Ye, 2019; Samal et al., 2019). This study represents the first instance of PFM applied to South Korean air pollution forecasting, an area desperately needing accurate air pollution measures due to the high levels of air pollution in South Korea.

### Purpose

Although PM_2.5_, PM_10_, O_3_, NO_2_, SO_2_, and CO clearly pose an imminent health and economic threat towards affected countries, effective machine learning models that account for seasonal variations, while making accurate long-term predictions have yet to be implemented in Seoul, South Korea. Therefore, in this research PFM was used for the accurate short-term and long-term forecasting of PM_2.5_, PM_10_, O_3_, NO_2_, SO_2_, and CO, which will allow for proper preparations to be made in advance of heavy air pollution. In addition to meeting this purpose, certain criteria must also be met to prove the efficacy of PFM. PFM must be able to interpolate values that are missing or are sensor errors. PFM must be scalable to other cities and countries. PFM also needs to have a low error and make predictions for the following year.

## Materials and Methods

### Locations of investigation and data sourcing

In this study, the dataset for Seoul air pollution was sourced from Seoul Open Data Plaza and the Seoul Metropolitan City Institute of Health and Environment. Air pollutant concentrations were recorded hourly for PM_2.5_, PM_10_, O_3_, NO_2_, SO_2_, and CO from 2017–2019.

### Data processing

The data consisted of 25 different zones in Seoul; however, the Jung district located in the center of Seoul was modeled with PFM and served as a model for Seoul air pollution. The 26,241 hourly values were manually processed on Excel to fit the input requirements for the model. The dates and hours were changed to a M-D-Y HH:MM:SS format to satisfy PFM’s formatting requirements. Any error reading from the sensors denoted by “-1” was replaced with “NA” as PFM would interpolate the missing values. PFM is able to interpolate missing values by fitting the model to nearby points allowing for the prediction of that value to replace the missing value.

### Facebook Prophet Forecasting Model (PFM)

PFM is a powerful tool for Python and R released in 2017 by Facebook to model time series datasets with trends, seasonality, and holidays (Taylor & Letham, 2017; Ye, 2019). PFM takes a few seconds to fit the model with tunable parameters. PFM is represented by the following formula:

(1) \begin{eqnarray*}y(t)=g(t)+s(t)+h(t)+{\epsilon }_{t}.\end{eqnarray*}

Equation (1) is the generalized prediction equation for PFM (1). The predicted value, *y*(*t*), is determined by the linear or logistic equation, *g*(*t*); seasonality based on yearly, monthly, daily, or another period of time, *s*(*t*); holiday outliers, *h*(*t*); and unexpected error, *ϵ*_*t*_ (1). The model has several parameters that can be optimized for better forecasting. The model type can be declared as linear or logistic. In linear models, outliers are normalized and there is no maximum or minimum limit set. Logistic models are used for saturated forecasts, where the highest and lowest values are specified. Either model is plausible but due to the outlier-filled datasets common with air pollution, linear models were used to ensure that PFM accounted for the typical outliers observed in air pollution trends.

Changepoints are another important parameter in PFM. Although there is an automatic function, the specific number of changepoints or scale of fit can be specified. The larger the changepoints, the better the model is at fitting the data; however, the model loses effectiveness at predicting future trends. When fewer changepoints are used, the model is underfitted and too general leading to the inability of an accurate graphical model. To determine the number of changepoints, PFM initially plots a large number and then uses L1 regularization to select the few points that will be used in the model. L1 regularization was used to select only the significant changepoints to keep the number of changepoints low to avoid overfitting.

(2) \begin{eqnarray*}L(x,y)\equiv \sum _{i=1}^{n}({y}_{i}-{h}_{\theta }({x}_{i}))^{2}+\lambda \sum _{i=1}^{n}\mid {\theta }_{i}\mid .\end{eqnarray*}

Equation (2) is the L1 regularization. x and y represent the coordinates of the changepoints.

${\mathop{\sum }\nolimits }_{i=1}^{n}({y}_{i}-{h}_{\theta }({x}_{i}))^{2}$ represents the loss function or the difference between the forecasted and actual value squared. By adding in the $\lambda {\mathop{\sum }\nolimits }_{i=1}^{n}\mid {\theta }_{i}\mid $, the weights will be smaller to avoid overfitting. In this formula, *λ* determines how much the weights are penalized. A low value results in a high bias and a high value results in underfitting, so a balance must be reached. PFM could determine the value for *λ* based on the average rate of change or the value can be specified based on the number of estimators.

**Figure 1:**
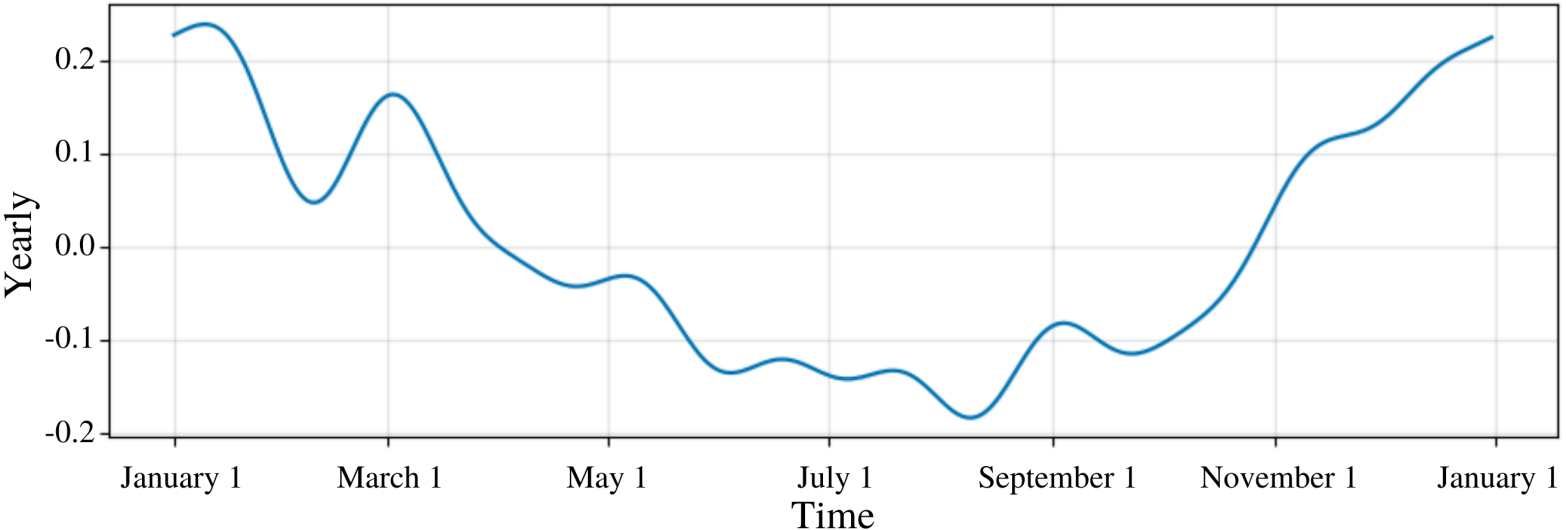
Yearly trend for CO. The graph is the predicted average graph of CO on a yearly basis. The periodicity of CO levels are shown as in December and January the CO concentrations are high. In July and August, CO concentrations decrease. The yearly seasonality for the model was used. Similar to this graph, overall trend, weekly, and daily graphs are also plotted.

Seasonality was another parameter that was important in forecasting new values. PFM offers plot components to show seasonality over daily, weekly, yearly, or lifetime intervals. Figure 1 shows CO’s yearly graph demonstrating the seasonality of CO concentrations reaching peaks at December and January and lows in July and August (Fig. 1). Any custom seasonality or holiday components could also be graphed.

Cross validation for the model was performed on a 40 day period to predict the error given historical data. Since 3 year data from 2017 to 2019 was used, the first two years (2017–18) were the initial training data. After training PFM with data from 2017-2018, the model predicted air pollution for the 2019 year and compared it to the actual values to determine PFM’s performance. PFM was then used to forecast air pollution in 2020.

### Statistical analysis

To analyze the models performance, several statistical values were calculated: Pearson’s Correlation Coefficient, Mean Squared Error (MSE), Root Mean Squared Error (RMSE), Mean Absolute Error (MAE), and coverage. Pearson’s Correlation Coefficient was used to determine whether the model was overfitted or underfitted. Values around 0.5 are ideal as the general trend is mapped, but the model is not overfitted. MSE is the average difference squared for the estimated and predicted value (3). RMSE is the square root of the MSE (4). MAE is calculated as the average difference between the predicted and actual values (5). RMSE places higher importance on outliers than MAE, making the difference between the two the impact of outliers on the data. Coverage determines the probability that the forecasted value will lie between the interval predicted by PFM; the default value for PFM is 80%. A higher coverage value represents lower variability in the dataset.

(3) \begin{eqnarray*}MSE= \frac{1}{n} \sum _{i=1}^{n}({y}_{i}-{\hat {y}}_{i})^{2}\end{eqnarray*}

(4) \begin{eqnarray*}RMSE=\sqrt{ \frac{1}{n} \sum _{i=1}^{n}({y}_{i}-{\hat {y}}_{i})^{2}}\end{eqnarray*}

(5) \begin{eqnarray*}MAE= \frac{1}{n} \sum _{i=1}^{n}\mid {y}_{i}-{\hat {y}}_{i}\mid \end{eqnarray*}

## Results

### PM_2.5_ modeling

**Figure 2:**
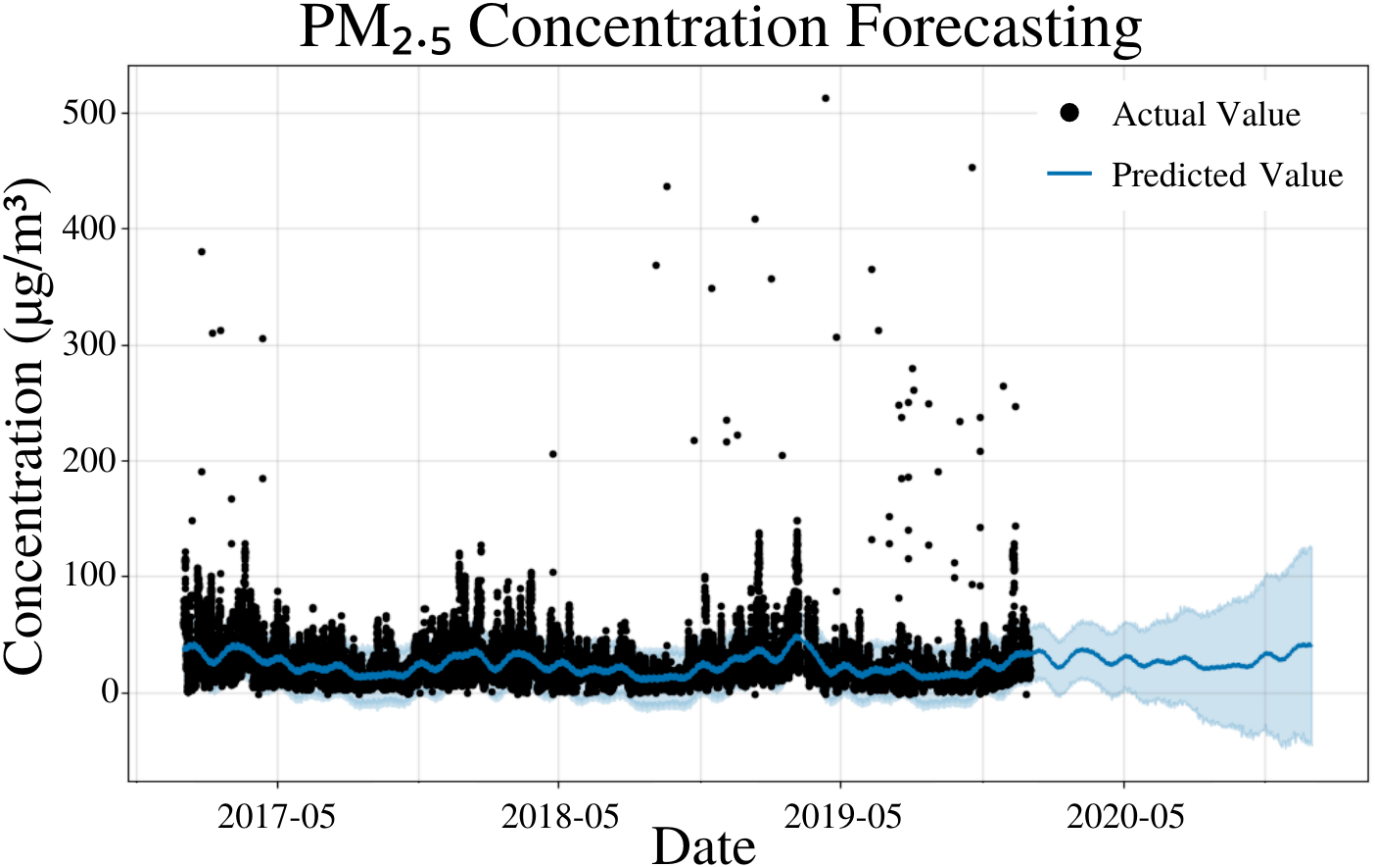
PM_2.5_ concentration forecasting in Deoksugung-gil, Jung-gu. The Prophet model’s predictions are compared to the actual values. The prediction for the 2020 year is shown.

To specify the features of PFM, a linear model was inputted; changepoints were determined using the L1 regularization method from PFM, and error was determined automatically by PFM (Fig. 2). Pearson’s correlation coefficient for the training years (2017–18) was 0.405, showing that the model was not overfitted for that interval. Also, the model was able to predict the entire 2019 year accurately, with only 2 years (2017–18) of data. This model is not overfitted due to its ability to predict 2019 PM_2.5_ concentrations effectively (Fig. 2). In all four statistical indicators (MSE, RMSE, MAE, coverage), short-term predictions were more accurate than long-term predictions. In a 5 day prediction, the MAE value was 12.6 µg/m^3^, while the RMSE value was 19.9 µg/m^3^, a difference of 7.3 µg/m^3^ (Table 1). The large RMSE and MAE difference (7.3 µg/m^3^) can be attributed to the outliers present in the dataset as RMSE places a larger weight on larger differences between values (Table 1). As prediction time increased from 5 days to 365 days, the MAE and RMSE increased by 4.2 µg/m^3^ and 5.9 µg/m^3^, respectively (Table 1). As prediction time increased from 5 days to 365 days, the coverage also decreased by 5.8% (Table 1). The increasing error and decreasing coverage as prediction time increases is due to the unpredictability of new trends that may occur in the future. Figure 2 and Table 1 demonstrate the efficacy of the model to predict future PM_2.5_ concentrations effectively.

**Table 1:** PM_2.5_ 2019 model performance statistics. The Prophet model forecasted 2019 values on an hourly basis, which was compared to actual values over a 5 day, 15 day, 1 month, and 1 year period. Units for MSE, RMSE, and MAE are µg/m^3^. The MAE was on average 12.6 µg/m ^3^ away from the actual value after 5 days and as the time increased away from the known values, the error increased.

Time	MSE	RMSE	MAE	Coverage
5 Days	3.96 × 10^2^	1.99 × 10^1^	1.26 × 10^1^	85.3%
15 Days	3.92 × 10^2^	1.98 × 10^1^	1.27 × 10^1^	84.4%
1 Month	4.09 × 10^2^	2.02 × 10^1^	1.29 × 10^1^	83.9%
6 Months	5.57 × 10^2^	2.35 × 10^1^	1.51 × 10^1^	81.0%
1 Year	6.73 × 10^2^	2.58 × 10^1^	1.68 × 10^1^	79.5%

### PM_10_ modeling

**Figure 3:**
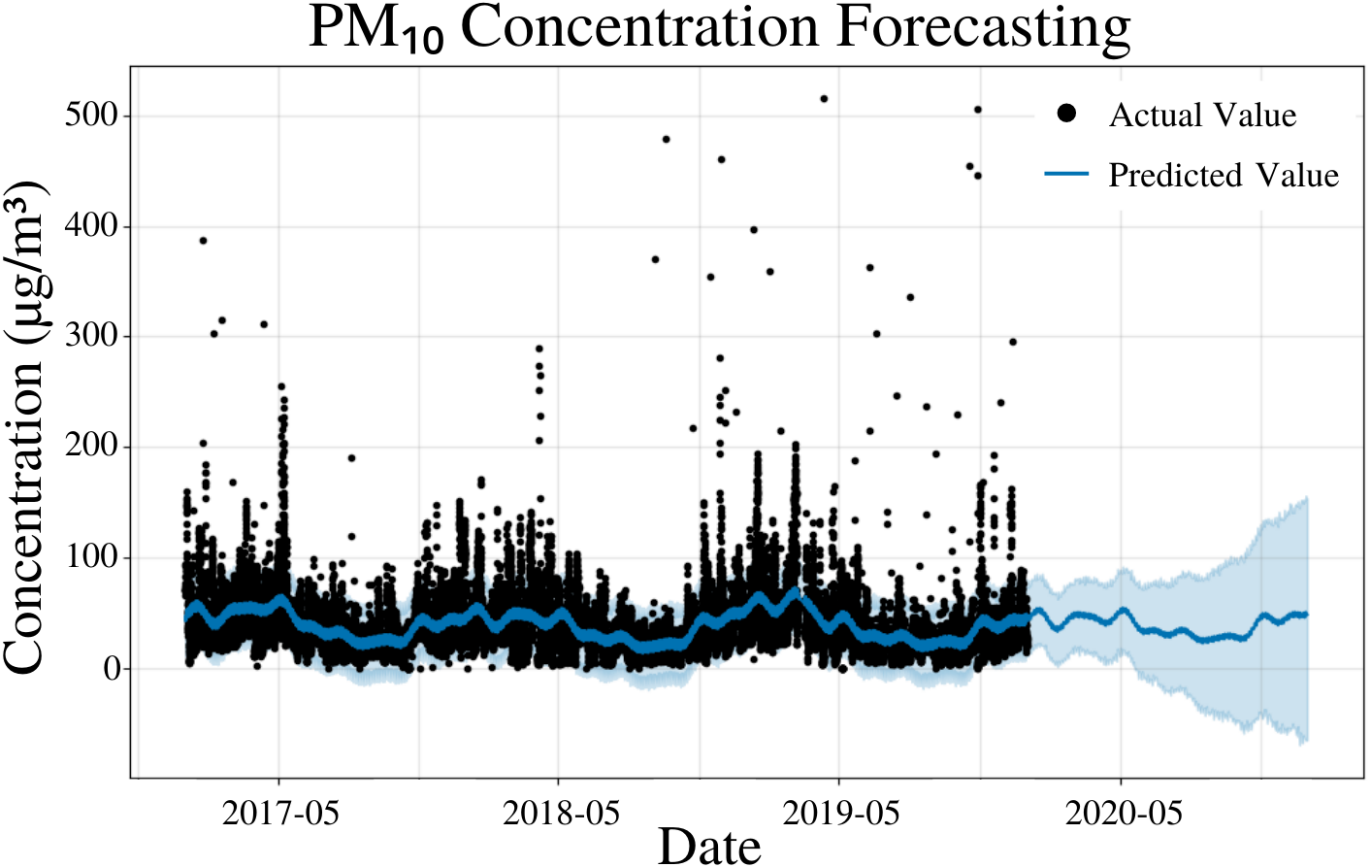
PM_10_ concentration forecasting in Deoksugung-gil, Jung-gu. The Prophet model’s predictions are compared to the actual values. The prediction for the 2020 year is shown.

To specify the features of PFM, a linear model was inputted, changepoints were determined using the L1 regularization method from PFM and error was determined automatically by PFM (Fig. 3). Pearson’s correlation coefficient for the training years (2017–18) was 0.446, showing that the model was not overfitted for that interval. Its ability to predict 2019 PM_10_ concentrations effectively (Fig. 3) also shows that the model was not overfit. All four statistical values (MSE, RMSE, MAE, and coverage) showed that short-term predictions were better than long term predictions. In a 5 day prediction, the MAE was 19.6 µg/m^3^ and the RMSE value was 29.3 µg/m^3^, 9.7 µg/m^3^ higher than the MAE value (Table 2). This difference between the MAE and RMSE (9.7 µg/m^3^) is attributed to the outliers and high variance in the dataset, a characteristic that is normal in air quality datasets (Table 2). As the prediction time increased from 5 days to 365 days, the MAE value increased by 7.6 µg/m^3^ (Table 2). As prediction time increased from 5 days to 365 days, the coverage of the data also decreased over the same interval from 79% to 74% (Table 2). The increasing error and decreasing coverage observed with increased prediction times is attributed to the erratic trends observed in air pollution. Figure 3 and Table 2 demonstrate the effectiveness of PFM for predicting PM_10_.

**Table 2:** PM_10_ 2019 model performance statistics. The Prophet model forecasted 2019 values on an hourly basis, which was compared to actual values over a 5 day, 15 day, 1 month, and 1 year period. Units for MSE, RMSE, and MAE are µg/m^3^. The MAE was on average 19.6 µg/m^3^ away from the actual value after 5 days and as the time increased away from the known values, the error increased.

Time	MSE	RMSE	MAE	Coverage
5 Days	8.60 × 10^2^	2.93 × 10^1^	1.96 × 10^1^	79.0%
15 Days	8.78 × 10^2^	2.96 × 10^1^	2.01 × 10^1^	77.1%
1 Month	8.98 × 10^2^	3.00 × 10^1^	2.03 × 10^1^	76.7%
6 Months	1.27 × 10^3^	3.55 × 10^1^	2.42 × 10^1^	77.6%
1 Year	1.67 × 10^3^	4.04 × 10^1^	2.72 × 10^1^	74.0%

### O_3_ modeling

**Figure 4:**
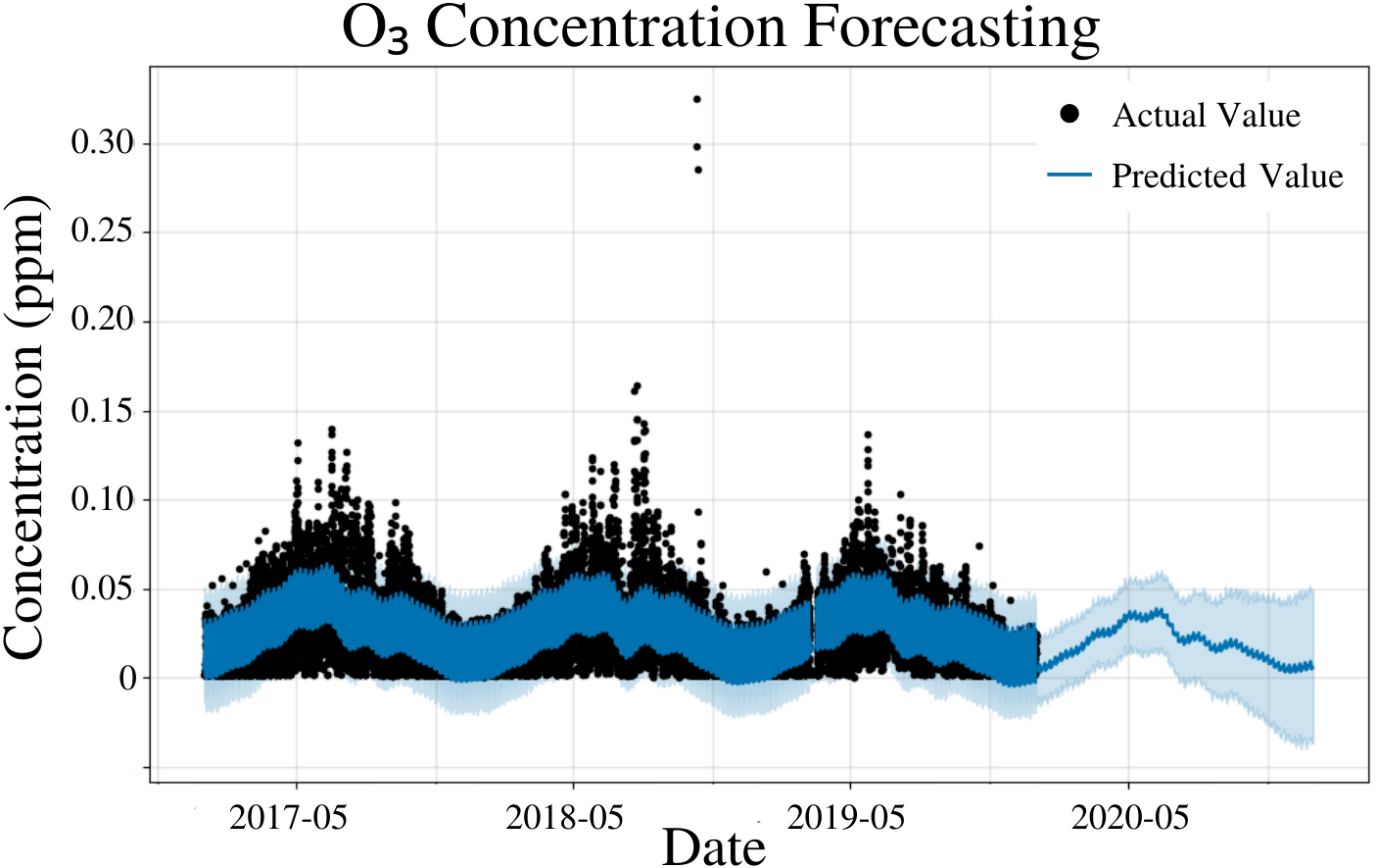
O_3_ concentration forecasting in Deoksugung-gil, Jung-gu. The Prophet model’s predictions are compared to the actual values. The prediction for the 2020 year is shown.

To specify the features of PFM, a linear model and yearly seasonality were inputted, the changepoint scale was experimentally determined to be 0.17 and error was determined automatically by PFM (Fig. 4). Pearson’s correlation coefficient for the training years (2017–18) was 0.6813, showing that the model was not overfitted for that interval. The value was higher than the other concentrations, due to the low variability of the data. Also, the model was able to predict the entire 2019 year accurately, with only 2 years (2017–18) of data. All 4 statistical measures (MSE, RMSE, MAE, and coverage) demonstrated that the model was better forecasting short-term rather than long-term predictions. In a 5 day prediction, the MAE was 0.0113 ppm and the RMSE was 0.0153 ppm, 0.004 ppm higher than the MAE value (Table 3). This difference between MAE and RMSE (0.004 ppm) demonstrates the prevalence of outliers and variance in the dataset for O_3_ values (Table 3). As prediction time increases from 5 days to 365 days, MAE and RMSE increase by 0.0021 ppm and 0.0024 ppm, respectively (Table 3). As prediction time increased from 5 days to 365 days, the coverage of the data also decreased by 4.4% (Table 3). Although these reductions in accuracy are minimal, they are expected as a result of the increased unpredictability as prediction intervals increase. Figure 4 and Table 3 demonstrates that PFM is extremely accurate over both short-term and long-term predictions for O_3_.

**Table 3:** O_3_ 2019 model performance statistics. The Prophet model forecasted 2019 values on an hourly basis, which was compared to actual values over a 5 day, 15 day, 1 month, and 1 year period. Units for MSE, RMSE, and MAE are ppm. The MAE was on average 0.0113 ppm away from the actual value after 5 days and as the time increased away from the known values, the error increased; the total MAE increase from 5 to 365 days was 0.0021 ppm.

Time	MSE	RMSE	MAE	Coverage
5 Days	2.34 × 10^−4^	1.53 × 10^−2^	1.13 × 10^−2^	80.9%
15 Days	2.47 × 10^−4^	1.57 × 10^−2^	1.15 × 10^−2^	80.3%
1 Month	2.58 × 10^−4^	1.60 × 10^−2^	1.17 × 10^−2^	79.9%
6 Months	2.99 × 10^−4^	1.73 × 10^−2^	1.30 × 10^−2^	78.5%
1 Year	3.13 × 10^−4^	1.77 × 10^−2^	1.34 × 10^−2^	76.5%

### NO_2_ modeling

**Figure 5:**
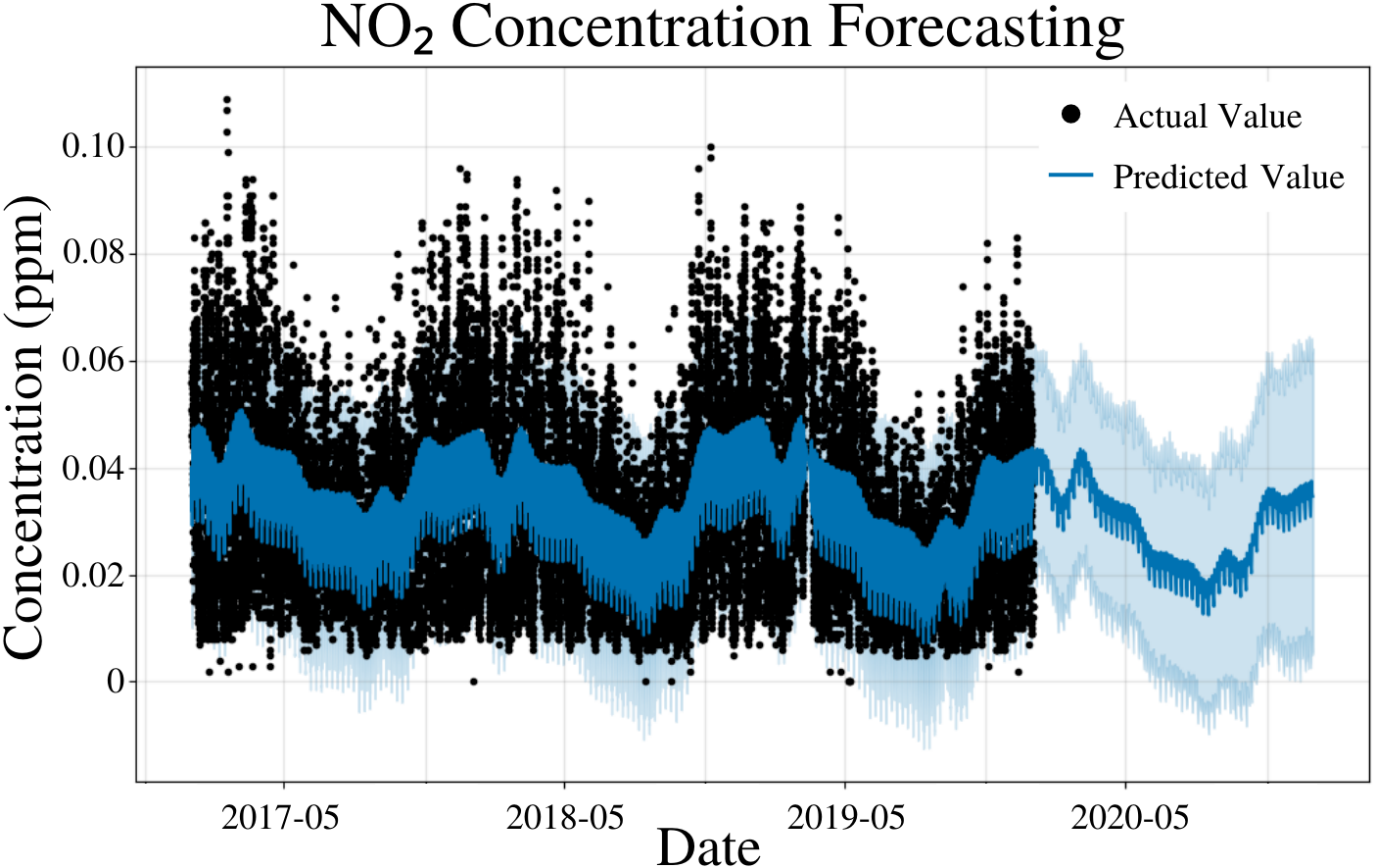
NO_2_ concentration forecasting in Deoksugung-gil, Jung-gu. The Prophet model’s predictions are compared to the actual values. The prediction for the 2020 year is shown.

To specify the features of PFM, a linear model, daily seasonality, and yearly seasonality were inputted, changepoints were determined using the L1 regularization method from PFM and error was determined automatically by PFM (Fig. 5). Pearson’s correlation coefficient for the training years (2017–18) was 0.432, showing that the model was not overfitted for that interval. Also, the model was able to predict the entire 2019 year accurately, with only 2 years (2017–18) of data. All 4 statistical measures (MSE, RMSE, MAE, and Coverage) demonstrated that the model was better forecasting short-term rather than long-term. In a 5 day interval, the MAE was 0.0128 ppm and the RMSE was 0.0163 ppm; the 0.0035 difference between RMSE and MAE is attributed to variance within the dataset (Table 4). As prediction time increased from 5 days to 365 days, MAE increased by 0.0001 ppm and RMSE decreased by 0.0001 ppm (Table 4). Despite this, coverage decreased by 1.3% as prediction time increased from 5 days to 365 days (Table 4). PFM’s relatively stagnant error values across numerous prediction intervals indicated that PFM is versatile for different prediction intervals. Figure 5 and Table 4 demonstrate that PFM performs effectively for both the short-term and long-term prediction of NO_2_.

**Table 4:** NO_2_ 2019 model performance statistics. The Prophet model forecasted 2019 values on an hourly basis, which was compared to actual values over a 5 day, 15 day, 1 month, and 1 year period. Units for MSE, RMSE, and MAE are ppm. The MAE was on average 0.0128 ppm away from the actual value after 5 days and as the time increased away from the known values, the error decreased and then increased.

Time	MSE	RMSE	MAE	Coverage
5 Days	2.64 × 10^−4^	1.63 × 10^−2^	1.28 × 10^−2^	79.0%
15 Days	2.57 × 10^−4^	1.60 × 10^−2^	1.27 × 10^−2^	79.5%
1 Month	2.53 × 10^−4^	1.59 × 10^−2^	1.26 × 10^−2^	79.7%
6 Months	2.54 × 10^−4^	1.59 × 10^−2^	1.26 × 10^−2^	78.7%
1 Year	2.64 × 10^−4^	1.62 × 10^−2^	1.29 × 10^−2^	77.7%

### SO_2_ modeling

**Figure 6:**
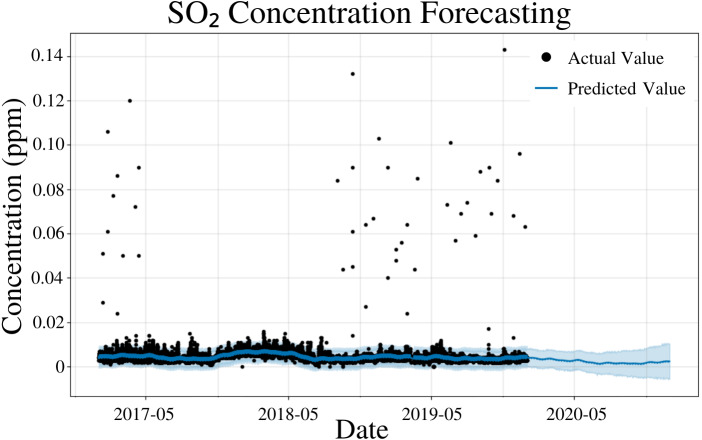
SO_2_ concentration forecasting in Deoksugung-gil, Jung-gu. The Prophet model’s predictions are compared to the actual values. The prediction for the 2020 year is shown.

To specify the features of PFM, a linear model and a weekly seasonality were inputted; changepoints were determined using the L1 regularization method from PFM and error was determined automatically by PFM (Fig. 6). Pearson’s correlation coefficient for the training years (2017–18) was 0.443, showing that the model was not overfitted for that interval. Also, the model was able to predict the entire 2019 year accurately, with only 2 years (2017–18) of data. All 4 statistical values (MSE, RMSE, MAE, and Coverage) demonstrated better predictions on a short term basis rather than a long term prediction. In a 5 day prediction, the MAE was 0.00124 ppm and the RMSE was 0.0027 ppm, only 0.00146 ppm higher than the MAE value (Table 5). The small difference between MAE and RMSE (0.00146 ppm) demonstrates the decreased prevalence of outliers and variance in the SO_2_ dataset (Table 5). As prediction time increases from 5 days to 365 days, MAE and RMSE increased by 0.00117 ppm and 0.00164, respectively (Table 5). Coverage also decreased by 9.8% as the prediction interval increased from 5 days to 365 days (Table 5). This increased error and decreased coverage is a result of unforeseeable conditions that may appear as the time progresses. Figure 6 and Table 5 demonstrates that PFM is able to effectively forecast SO_2_ concentrations up to a year in advance.

**Table 5:** SO_2_ 2019 model performance statistics. The Prophet model forecasted 2019 values on an hourly basis, which was compared to actual values over a 5 day, 15 day, 1 month, and 1 year period. Units for MSE, RMSE, and MAE are ppm. The MAE was on average 0.00124 ppm away from the actual value after 5 days and as the time increased away from the known values, the error increased.

Time	MSE	RMSE	MAE	Coverage
5 Days	7.39 × 10^−6^	2.70 × 10^−3^	1.24 × 10^−3^	94.5%
15 Days	1.03 × 10^−5^	3.20 × 10^−3^	1.37 × 10^−3^	92.1%
1 Month	1.06 × 10^−5^	3.24 × 10^−3^	1.40 × 10^−3^	91.5%
6 Months	1.57 × 10^−5^	3.93 × 10^−3^	2.00 × 10^−3^	86.6%
1 Year	1.92 × 10^−5^	4.34 × 10^−3^	2.41 × 10^−3^	84.7%

### CO modeling

**Figure 7:**
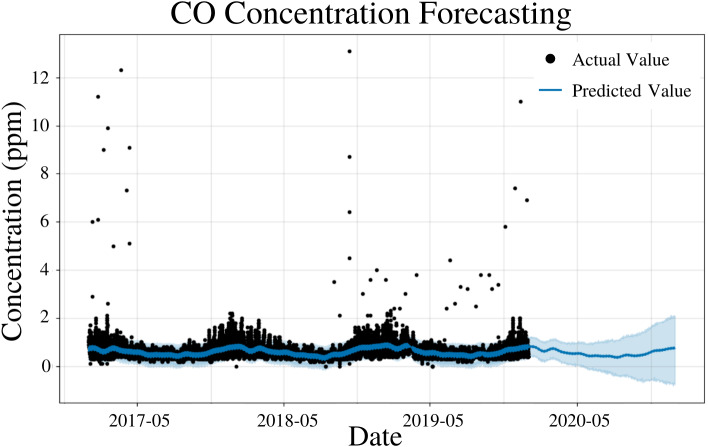
CO concentration forecasting in Deoksugung-gil, Jung-gu. The Prophet model’s predictions are compared to the actual values. The prediction for the 2020 year is shown.

To specify the features of PFM, a linear model and a weekly seasonality were inputted, changepoints were determined using the L1 regularization method from PFM and error was determined automatically by PFM (Fig. 7). Pearson’s correlation coefficient for the training years (2017–18) was 0.406, showing that the model was not overfitted for that interval. Also, the model was able to predict the entire 2019 year accurately, with only 2 years (2017–18) of data. All 4 statistical measures (MSE, RMSE, MAE, and Coverage) demonstrated that the model was better forecasting short-term rather than long-term predictions. In a 5 day prediction, the MAE was 0.207 ppm and the RMSE was 0.308 ppm, 0.101 ppm higher than the MAE value (Table 6). The 0.101 ppm difference between the RMSE and MAE demonstrates the relatively high prevalence of outliers and variance in the dataset (Table 6). As prediction time increases from 5 days to 365 days, MAE and RMSE increase by 0.18 ppm and 0.237 ppm, respectively (Table 6). As prediction time increased from 5 days to 365 days, the coverage of the data also decreased by 14.2% (Table 6). This demonstrates that although PFM can still predict for CO over long prediction intervals, PFM may be better suited for shorter predictions as the error increases significantly as the prediction interval increases. Figure 7 demonstrates that PFM is extremely accurate for short-term predictions and relatively accurate for long-term predictions for CO.

**Table 6:** CO 2019 model performance statistics. The Prophet model forecasted 2019 values on an hourly basis, which was compared to actual values over a 5 day, 15 day, 1 month, and 1 year period. Units for MSE, RMSE, and MAE are ppm. The MAE was on average 0.207 ppm away from the actual value after 5 days and as the time increased away from the known values, the error increased.

Time	MSE	RMSE	MAE	Coverage
5 Days	9.47 × 10^−2^	3.08 × 10^−1^	2.07 × 10^−1^	86.9%
15 Days	1.12 × 10^−1^	3.34 × 10^−1^	2.11 × 10^−1^	85.7%
1 Month	1.25 × 10^−1^	3.52 × 10^−1^	2.21 × 10^−1^	83.8%
6 Months	2.42 × 10^−1^	4.83 × 10^−1^	3.28 × 10^−1^	72.2%
1 Year	3.07 × 10^−1^	5.45 × 10^−1^	3.87 × 10^−1^	72.7%

## Discussion

Although PFM is only trained with 2 years (2017-2018) of data and tested on one year (2019) of data, the results compare favorably to similar studies. Previous literature has proposed numerous methods to accurately predict PM_2.5_ concentrations (Xi et al., 2015; Deters et al., 2017; Li et al., 2015; Scher & Messori, 2019; Maleki et al., 2019; Pasero & Mesin, 2010). Ye (2019) utilized a hybrid ARIMA-PFM model to generate a 2 day forecast of air pollutants in Shenzhen. The ARIMA-PFM model had an MSE of 107.83 µg/m^3^ for PM_2.5_, much less than our MSE of 396 µg/m^3^ for PM_2.5_ indicating that our dataset contains more outliers (Ye, 2019). Still, our model had nearly 1/2 the MAE at 11.2 µg/m^3^ than the ARIMA-PFM model’s MAE at 23.75 µg/m^3^ (Ye, 2019). Despite our datasets high variance, PFM was still able to demonstrate a lower MAE than previous models indicating our models favorability compared to others.

PFM compared favorably to other models developed for PM_10_ prediction. Sayegh et al. compared 5 different models for PM_10_ prediction: Generalised Additive Model (GAM), Multiple Linear Regression Model (MLRM), Quantile Regression Model (QRM), and Boosted Regression Trees 1-way (BRT1) and 2-way (BRT2). GAM, MLRM, QRM, BRT1, and BRT2 exhibited MAE values of 74.3 µg/m^3^, 80.0 µg/m^3^, 61.0 µg/m^3^, 75.6 µg/m^3^, and 80.4 µg/m^3^, respectively, which was 3.7 times, 4 times, 3.1 times, 3.86 times, and 4.1 times, our model’s MAE of 19.6 µg/m^3^ (Sayegh, Munir & Habeebullah, 2014). Ye 2019 proposed another comparable hybrid PFM-ARIMA model demonstrating an MSE of 240.73 µg/m^3^, less than our MSE of 860 µg/m^3^ (Ye, 2019). This high MSE demonstrates the variance found in the Seoul dataset. Despite this, our MAE (19.6 µg/m^3^) was still comparable with Ye 2019’s MAE (19.5 µg/m^3^) (Ye, 2019). Despite the high variance in the Seoul dataset, PMF was still able to perform comparably with other models that used datasets with less variance.

PFM compared favorably to comparable models for O_3_ prediction. Hadjiiski & Hopke (2000) proposed the use of an Artificial Neural Network with meteorological parameters. ANNs exhibited a 5 h RMSE value of 0.01146 ppm, while our PFM had a 5 h RMSE of 0.1043 ppm (Hadjiiski & Hopke, 2000). This demonstrates the high variance observed in the O_3_ dataset. Ye 2019’s hybrid PFM-ARIMA model produced a MAE of 0.012 ppm (2.36 µg/m^3^), 0.001 ppm less than our PFM, which had an MAE of 0.013 ppm (Ye, 2019). Despite our dataset’s high variance as demonstrated by PFM’s high RMSE values, our MAE was still able to remain low and comparable to similar studies.

PFM compares favorably to other NO_2_ forecasting models. Capilla et al. developed 16 Levenberg–Marquardt algorithm models to predict NO_2_ concentrations; the lowest MAE from these 16 models was 0.0137 ppm (Longhurst et al., 2015). PFM’s MAE value was the lowest among all of these values with 0.0128 ppm (Longhurst et al., 2015). This demonstrates that PFM performed more accurately than the models proposed by Capilla et al. Furthermore, Ye (2019) developed a hybrid ARIMA-PFM model that exhibited a MAE of 0.013 ppm (24.68 µg/m^3^), 0.0002 ppm higher compared to PFM with a MAE of 0.0128 ppm. PFM’s prediction of NO_2_ was in most cases better than are comparable to previous models demonstrating PFM’s efficacy for air pollution prediction.

Our model for SO_2_ compares favorably to other models. Shaban, Kadri & Rezk (2016) used M5P model trees (M5P), Support Vector Machine (SVM), and Artificial Neural Network (ANN) to forecast the next day’s data. The RMSE for the M5P, SVM, and ANN values were 0.0588 ppm, 0.0995 ppm, and 0.1076 ppm, respectively, which was 2.2 times, 3.7 times, and 4.0 times, our model’s RMSE at 0.0270 ppm (Shaban, Kadri & Rezk, 2016). Another model developed by Ye 2019 using a hybrid ARIMA-PFM model demonstrated similar results as our model was more than 5 times as accurate as the Ye 2019’s model (Ye, 2019). model had an average of MAE of 0.006587 ppm (18.58 µg/m^3^) more than 5 times our MAE at 0.00124 ppm (Ye, 2019).

PFM’s ability to forecast CO is very similar to other models proposed in previous literature. Ye 2019 developed a hybrid ARIMA-PFM that has an RMSE of 0.009 ppm, nearly 1/3 our RMSE of 0.308 ppm (Ye, 2019). This indicates that Ye 2019’s dataset has less variance than the one used in this study. Despite this, Ye 2019’s model had a MAE of 10.274 ppm (11.77 µg/m^3^), nearly 50 times our MAE of 0.207 ppm (Ye, 2019). This indicates that our model is significantly more accurate than Ye 2019’s model despite the variance in our dataset.

## Conclusions

The accurate prediction of air quality, both long-term and short-term, is a crucial step in mitigating the damage caused by air pollution. This allows government organizations to prepare for air pollution-induced economic and health repercussions by predicting the period of time in which air pollution may be especially high. An accurate timeline can be constructed to give lawmakers an idea of when actions need to be taken. This allows government organizations and businesses to prepare for any economic or health disturbances caused by air pollution-related phenomena. PFM’s predictions in this study also allow millions of civilians in Seoul, South Korea to properly prepare for days in which air pollution is dangerously high. This research demonstrates PFM’s unique ability to accurately forecast both short-term and long-term air pollution with regards to seasonality, an important variable with air pollution modelling that ordinary prediction models (Random Forest Regression, Linear Regression, etc.) fail to address. Although PFM has been used to model air pollution in a few research studies, it’s applications are still relatively unexplored in the field of environmental modelling (Ye, 2019; Samal et al., 2019). Furthermore, its application across different countries has yet to be explored.

Despite PFM’s ability to accurately forecast both short-term and long-term air pollution in Seoul, several limitations exist. The data published by Seoul Open Data Plaza only accounted for 3 years of data (2017–2019). This restricted us from making accurate predictions into 2021 due to the lack of available data. Although PFM’s seasonality accounts for some aspects of meteorology, PFM does not explicitly account for meteorological events. This means that significant weather events may cause outliers that PFM is unable to account for. However, this was intentional due to the restrictions that meteorological parameters place on the versatility of the model’s prediction window.

This research illustrated PFM’s ability to predict air pollution in Seoul, South Korea. PFM is not regionally specific to Seoul, South Korea and can be applied to other regions due to PFM’s lack of complex variables and fast training time (approximately 10 times faster than ARIMA); therefore, making PFM scalable (Ye, 2019). Therefore, future research should be conducted to apply PFM to other regions in which air pollution poses an imminent threat, such as Beijing, China and Los Angeles, California. Furthermore, despite this studies emphasis on air pollution, PFM should also be applied as a prediction technique to other fields, such as environmental economics, to forecast how various economic markets will be impacted amidst the impending climate change crisis.

## Additional Information and Declarations

### Data Availability

The following information was supplied regarding data availability: The data sourced from Seoul Open Data Plaza is available in FigShare: Valagolam, Davesh; Shen, Justin (2020): Air Pollution Data for Seoul. figshare. Dataset. https://doi.org/10.6084/m9.figshare.12805643.v2.
